# Full life cycle test with *Eisenia fetida* - copper oxide NM toxicity assessment

**DOI:** 10.1016/j.ecoenv.2022.113720

**Published:** 2022-08

**Authors:** J.J. Scott-Fordsmand, A. Irizar, M.J.B. Amorim

**Affiliations:** a Department of Bioscience, Aarhus University, Vejlsøvej 25, DK-8600 Silkeborg, Denmark; b Department of Biology & CESAM, University of Aveiro, 3810–193 Aveiro, Portugal

**Keywords:** CuONM, Environment, Earthworms, Population effect, Life stages

## Abstract

Copper oxide nanomaterials (CuONM) are widely used, e.g. as antimicrobial coatings, wood preservatives,fertilizers, etc. Life cycle aspects of Copper Nanomaterials (CuONM) toxicity have been scarcely studied in earthworms, as the focus has been on standard survival and reproduction toxicity tests. Standard tests do not allow for an understanding of which life cycle stage is the most sensitive, and how this can be impacted by CuONMs toxicity to cause longer term population level effects. Since CuONM may release free Cu ions (Cu^2+^) it is relevant to compare CuONM toxicity with CuCl_2_ salt. Therefore, the aim of the present study was to assess the effects of CuONM and CuCl_2_ throughout the different stages of the full life cycle (FLC) of *Eisenia fetida* while comparing it with the OECD standard test. Additional endpoints included juvenile survival, growth, maturation, besides survival and reproduction. The FLC test showed that e.g. juvenile survival was especially susceptible during the first 28 days post-hatching, neither juvenile growth nor time to maturity were related to exposure concentration. Both CuONM and CuCl_2_ caused a concentration-dependent decrease in population growth, while a hormesis effect was observed at low concentrations of CuCl_2_. A reduction in instantaneous growth rate was observed at higher concentrations. The FLC test and the OECD test had different exposure history therefore the ECx values are not comparable across the test systems. Hence, the FLC test enabled the detection of the most vulnerable developmental stages and elucidate different life stage sensitivities between the two Cu exposures.

## Highlights

• Current OECD standard test miss specificity in the life stage evaluation.
• A specific life cycle (LC) test will provide a better environmental hazard assessment.
• A LC test shows that life stages are affected by various Cu forms.
• Different effects from LC include impact on hatching vs juvenile mortality.

## Graphical Abstract

**Figure:**
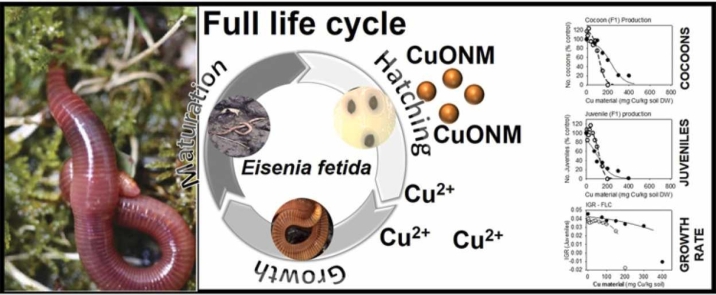

## 1 Introduction

The earthworms toxicity test is recommended by several international toxicity guidelines (ISO, 2008, OECD, 1984) due to the earthworm’s environmental importance and sensitivity against a broad type of compounds, including nanoparticles (Gomes et al., 2015a, Jensen et al., 2007, Joko et al., 2021, Mariyadas et al., 2018, Schnug et al., 2014). While the standard test provides information on survival, growth, and reproduction after 28/56-days exposure, these tests focus primarily on “one” life stage of the earthworm’s life cycle, i.e., effects after maturity is reached. Hence, the tests do not provide detailed information to elucidate the possible effects at the population level. In various other worm species, it is shown that adulthood is not always the most sensitive life-stage, as juveniles’ stage can be more sensitive for various compounds (Bicho et al., 2015, Bindesbol et al., 2007, Spurgeon et al., 1994). It is true that the standard test also provides information on juvenile survival, i.e., counting after 56 days, but it is impossible to discriminate between cocoon production, hatching, and juvenile growth. The advantage of assessing toxicity during the full life cycle (FLC) of animals is that it allows identification and assessment of the impact at the different developmental stages (Bicho et al., 2016, Bicho et al., 2017a, Bicho et al., 2017b, Bicho et al., 2020, Koelmans et al., 2015, Kumar et al., 2014, Scott-Fordsmand et al., 2014, Scott-Fordsmand et al., 2017). There are not many species for which this is done, especially in the terrestrial environment, one example includes *Enchytraeus crypticus* where a FLC test has been developed (Bicho et al., 2015). This *E. crypticus* test is based on the exposure of the animals starting from their initial stage (cocoon) until they reproduce an F1 generation. During the life of the animals, relevant toxicity endpoint times were defined at different developmental stages, allowing to identify the most sensitive life-stages. Such additional endpoint data allows to model population level effects, where cocoon production, instantaneous growth rate or population growth can be estimated. Population modelling can show prediction in time, and hence the sustainability level for future generations.

Therefore, the aim of the present study was to use a FLC and multi-endpoint approach with *E. fetida* to test the toxicity of CuONM and compare it with the ionic CuCl_2_. Further, the OECD reproduction test was also performed in parallel, aiming to compare the sensitivity. Copper oxide nanomaterials (CuONM) were selected as the test material based on their wide usage, e.g. as antimicrobial, coatings, antifouling paints, catalysts, superconductors, thermo-electrics, sensing materials, wood preservatives and fertilizers. In addition to this widespread use of CuONM, the current toxicity research progress of CuONM is also needed, making this study meaningful. The present case study involves CuONM used in paint applications, which provides aesthetic functionality to softwood cladding in addition to wood preservation.

## 2 Experimental

### 2.1 Test material and characterization

Copper oxide nanomaterials (CuONM) (for details see Table 1) and copper chloride anhydrous (CuCl_2_, >99.9% purity, Acros Organics, Thermo Fisher Scientific, Geel, Belgium) were used.

**Table 1:** Summary of the main properties of the tested CuONM, for further information see (Mendes et al., 2018).

Properties	CuONM
Manufacturer	Plasma Chem
CAS number	1317–38‐0
Primary size distribution (average) [nm]	3–35 (12)
Mode (1st quartile - 3rd quartile) [nm]	10 (9.2–14)
Shape	Semi-spherical
Average crystallite size [nm]	9.3
Crystallite phases (%)	Tenorite 100%
Dispersability in water: D50 [nm];	139.5 ± 4.6;
Average agglomeration number (AAN)	346
Dispersability in modified MEM: D50 [nm];	85.2 ± 2.7;
Average agglomeration number (AAN)	77
Z‐potential in UP water [mV]	+ 28.1 ± 0.6
Isoelectric point [pH]	10.3
Photocatalysis: photon efficiency [unitless]	1.5 × 10–4
Specific Surface Area [m2/g]	47.0 ± 1.7
Pore sizes [nm]	13.5 ± 1.6 (BJH)
	23.0 ± 0.9 (AVG)
Surface chemistry [atomic fraction]	Cu = 0.46 ± 0.05; O = 0.47 ± 0.05
	C = 0.07 ± 0.01
Chemical impurities [mg/kg]	Na: 505 ± 30; Pb: 36 ± 2 Ag: 13 ± 4

### 2.2 In situ characterisation

Copper was measured in the soil, in soil-solution and organisms following the method details as in Gomes et al. (2015b) and Mendes et al., 2018 at sampling days 28, 56 and 84. In short, the total Cu was measured in soil, soil solution and worm tissue (by Graphite Furnace Atomic Absorption Spectroscopy: AAS-GF, Perkin Elmer 4100, Ueberlingen, Germany) and in soil solution the free active form was also measured (by ion-selective electrode ISE25Cu-9, with a REF251 reference electrode (Radiometer Analytical, Lyon, France)). For Cu measurements, all the samples were acid digested (HNO_3_, 65%) for 3–7 days on a hot plate with temperatures up to 110 ºC until all brown fumes were gone. Samples were re-dissolved in 2% HNO_3_ for analysis on the AAS. A reference tissue for copper concentration was used (National Institute of Standards and Technology, 1989). The amount of CuO present as nanomaterials in the soil was not determined, due to the technical difficulties/impossibilities i.e. particle size is below the detection limit of 15 nm as attempted (Navratilova et al., 2015).

### 2.3 Soil and spiking procedures

The exposures were performed in the standard natural soil LUFA 2.2 purchased from LUFA-Speyer (Germany). Soil was first dried for 24 h at 90 °C. Cu materials were added to obtain a final Cu concentration of 0, 75, 100, 150, 200, 300, 400, 500, 600 and 800 mg Cu/ kg soil dry weight (DW) or 0, 2.5, 5, 10, 20, 30, 40, 60, 80, 100, 150 and 200 mg Cu/ kg soil DW of CuCl_2_. The concentration ranges differed between the two exposures to optimise concentration response curves in each case [based on priori range finding experiment]. For CuONM, the powder was added to the dry soil, mixed and moisture was adjusted to 50% of the water holding capacity (WHC) with deionized water. In the case of CuCl_2_, serial dilutions of CuCl_2_ in distilled water were prepared and added to the soils to adjust their moisture (50% of WHC). Each concentration was replicated twice, each containing 500 g of dried soil.

### 2.4 Test species

Eisenia fetida earthworms were purchased from ECT Oekotoxikologie GmbH (Germany) and were maintained under culture conditions of 20 ± 1 °C, 12/12 h light/dark light regime and fed with cow dung.

### 2.5 Cocoon synchronization

In order to get enough juveniles for the full life cycle experiment, to start at the same time, the cocoons were synchronized. Synchronized cocoons were obtained by placing clitellate *Eisenia fetida* earthworms in a box with clean soil for 3 weeks. After this period of time, the cocoons were searched and hand collected, classified according to their developmental stage by their colour: yellow as cocoons in the early developmental stage and reddish in the late developmental stage. Yellow cocoons were placed in a plastic vessel with a thin layer of clean moist soil while the reddish cocoons were transferred to a petri dish with filter paper moist with Lumbricus Balanced Salt Solution (LBSS). The development of the yellowish cocoons was checked every two days and as they turned to reddish they were transferred to a petri dishes prepared as previously described. The hatching of the cocoons in the petri dishes was checked daily, and the hatchlings were maintained on petri dishes with filter paper moist with LBSS for a maximum of 3 days before the exposure. Pools of 10 hatchlings were weighted and transferred to plastic vessels containing 500 g dried soil.

### 2.6 Full life cycle test

The exposures were performed at a constant temperature of 20 °C ± 1 in a light regime of 16/8 h light/dark. Once a week, the water lost due to evaporation was replaced and the earthworms were fed with 5 g of cow manure until they reached maturity and with 10 g during the rest of the experiment.

The survival and growth of the earthworms were recorded every two weeks until 100% of the individuals exposed to all the concentrations reached sexual maturity (developed clitellum). After 77 days all juveniles reached maturity (see results section, Fig. 2d) and at this date the reproduction test was started, i.e., day 77 of growth, and the test was finished 56 days later, i.e., day 133, assessing survival and reproductive output.

The experiment was performed as explained below (see 2.7) and the results of this test were labelled as “Full Life-cycle test” in the paper.

### 2.7 Standard earthworm reproduction test

The reproduction test was performed following the Earthworm Reproduction test (OECD, 2004) with some modifications. Briefly, 10 clitellate worms weighing more than 250 mg were introduced in each vessel containing 500 mg dry weight contaminated with different CuONM and CuCl_2_ concentrations (see 2.3). The exposure was performed at a constant temperature of 20 °C ± 1 in a light regime of 16/8 h light/dark. Once a week, the water loss due to evaporation was replenished and the worms were fed with 10 g of cow manure. After 28 days, the worms were searched and removed, and the mortality and weight were recorded. The animals were then depurated for 24 h in a Petri dish with wet filter paper, frozen in liquid nitrogen and stored at − 80ºC for later metal tissue analysis. The cocoons were counted and exposed for 28 days more in the same conditions but without weekly food addition, and after the additional 28 days (56 days from the start of the experiment) soils were wet sieved and juveniles were quantified.

### 2.8 Individual growth rate, Instantaneous growth rate (IGR) and population growth

Individual growth rates were calculated from the models describing the growth curves of each treatment, which were adjusted using SigmaPlot program. The b parameter of the models was used as predictor of the growth rate. The Instantaneous growth rate (IGR) is an indicator of population growth, and it was calculated for juveniles and cocoons following the equation (ri): ri = ln(Nf/N0)/t, where Nf is the number of juveniles or cocoons at the end of the experiment, N0 is the initial number, and t is the duration of the experiment (Sibly, 1999). The population growth was calculated also at different developmental stages of the life-cycle test as the sum of juveniles, adults, and the produced juveniles.

### 2.9 Data analysis

The response and effect concentrations (EC10, EC50 and EC90 values) of CuONM and CuCl_2_ regarding survival, cocoon production, hatchability and juvenile production were estimated using 2 parameter logistic models in the Toxicity Relationship Analysis Program (TRAP 1.02). Parameter logistic models were built to describe the growth of earthworms performed in SigmaPlot 11.0. The Spearman’s correlation analysis was performed between the model parameters, survival, and Cu concentration to test the relationship between those variables (p < 0.05).

## 3 Results

### 3.1 Test materials – in situ characterisation

Measured total soil Cu concentration was ca. 92 ± 15% and 97 ± 30% (of the nominal) for CuONM and CuCl_2_, respectively. The soil solution contained less than 0.211 ± 0.008% of the total soil Cu for both CuONM and CuCl_2_. The Cu in soil solution (total and free ion) increased with time, especially at the highest CuONM concentration. The active Cu in soil solution (i.e., Cu^2+^) constituted less than 0.007% in both exposures, but the total Cu in the soil-solution was 2 − 3 fold higher for CuCl_2_ than for CuONM spiked soils.

### 3.2 Full Life-cycle test

The survival decreased in a concentration-dependent manner after exposure to CuONM and CuCl_2_ (Table 2, Fig. S1). CuCl_2_ exposure was more toxic to earthworms than CuONM, with a mass based 5 times lower LC50 (317 mg Cu/kg soil DW for CuONM vs 85 mg Cu/kg soil DW for CuCl_2_, Table 2).

**Table 2:** Effect Concentrations (ECx) estimated values (mg Cu/kg soil DW) of CuCl_2_ and CuONM for survival, reproduction and population growth in the full life cycle and in the OECD test. The 95% confidence interval is shown in brackets and the models and parameters are specified.

Endpoint	Cu material	EC10	EC50	EC90	Model & parameters
Full Life Cycle test					
Juvenile Survival	CuONM	207 (139–275)	317 (285–350)	428 (354–502)	Log 2 par; Y0:93.3; S: 2.3E-03;
	CuCl_2_	23 (−22 to 68)	85 (58–112)	147 (82–211)	Log 2 par; Y0:83.3; S: 5.53E-03;
Juvenile Growth	CuONM	No monotonic effect	No monotonic effect	No monotonic effect	–
	CuCl_2_	No monotonic effect	No monotonic effect	No monotonic effect	–
Time to Maturity	CuONM	No monotonic effect	No monotonic effect	No monotonic effect	–
	CuCl_2_	No monotonic effect	No monotonic effect	No monotonic effect	–
Adult Survival	CuONM	No effect	No effect	No effect	–
	CuCl_2_	No effect	No effect	No effect	–
Cocoon production	CuONM	92 (7–177)	229 (181–278)	367 (255–477)	Log 2 par; Y0:96.3; S: 2.7E-03;
	CuCl_2_	47 (11–83)	114 (91–137)	181 (129–233)	Log 2 par; Y0:120; S: 6E-03;
Cocoon Hatchability	CuONM	265 (184–346)	358 (320–397)	452 (372–532)	Log 2 par; Y0:98; S: 2.2E-03;
	CuCl_2_	No effect	No effect	No effect	–
Juvenile production	CuONM	62 (21–103)	118 (92–144)	174 (117–231)	Log 2 par; Y0:108; S: 6.8E-03;
	CuCl_2_	47 (12–83)	114 (91–138)	181 (129–233)	Log 2 par; Y0:120; S: 6E-03;
Population Growth	CuONM	n.d.	110 (75–118)	254 (164–346)	Log 2 par; Y0:120; S: 1.4E-03;
	CuCl_2_	60 (21–100)	117 (92–142)	174 (119–229)	Log 2 par; Y0:108; S: 6.7E-03;
Instantaneous Growth Rate	CuONM	59 (−82 to 200)	406 (226–585)	620 (363–878)	Log 2 par; Y0: 4.56E-02; S: 1.04E-03;
	CuCl_2_	108 (90–125)	173 (156–190)	238 (195–281)	Log 2 par; Y0: 3.7E-02; S: 3.4E-03;
OECD standard test					
Survival	CuONM	378 (134–624)	866 (623–1101)	1352 (772–1933)	Log 2 par; Y0:100; S: 2.5E-03;
Cocoons	CuONM	–	117 (72–161)	283 (155–411)	Log 2 par; Y0:100; S: 2,25E-03;
	CuCl_2_	–	93 (20–166)	189 (7–371)	Log 2 par; Y0:56; S: 4,85E-03;
Reproduction (juveniles production)	CuONM	12 (−106 to 131)	97 (39–154)	181 (35–327)	Log 2 par; Y0:98; S: 2E-03;
	CuCl_2_	n.d.	107 (−26 to 240)	223 (−119 to 565)	Log 2 par; Y0:111; S: 9E-03;

The exposed juveniles grew following a sigmoidal logarithmic curve in all the exposures (Fig. S2). CuONM and CuCl_2_ did not cause any clear monotonic concentration-dependent effect on the growth nor was this the case for the maximum weight of the animals (Fig. 1a & b, Table 2). However, the Spearman correlation analysis showed a significant relationship between the maximum weight and the survival rate of animals after exposures to CuONM and CuCl_2_ (r_s=_ −7536 for CuONM and r_s=_ −8883 for CuCl_2_: p < 0005) (Fig. 2c).

**Fig. 1:**
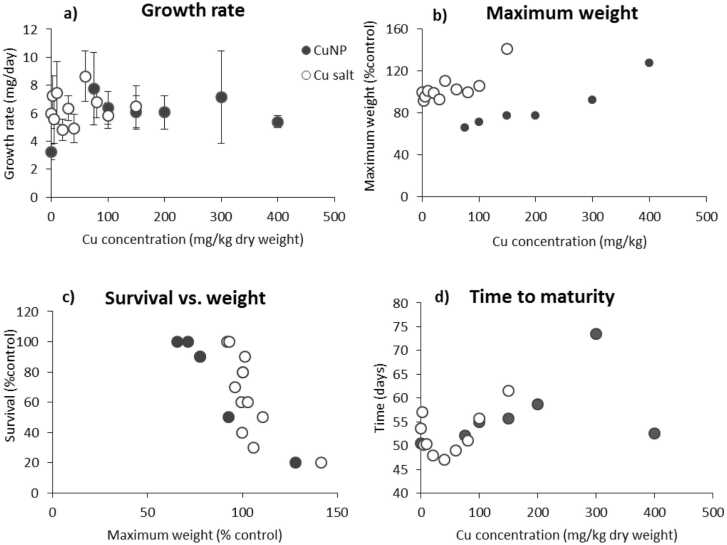
Parameters describing the growth and sexual development of *E. fetida* exposed to different concentrations (mg Cu/kg DW) of CuONM (10 concentration) and CuCl_2_ (12 concentrations), concentrations reported as, and the time needed to reach maturity (days) observed at 56 days of exposure. (a) The growth rate of the animals (mg/day). (b) Maximum weight of the earthworms. (c) relationship between survival (%control) and maximum weight of earthworms (%control). (d) the time to reach maturity (days). The growth rate and maximum weight of the animals were obtained from the logistic models. Symbols: closed/black circles refer to CuONM and open/white circles to CuCl_2_.

Maturity (defined based on visible clitellum) was reached between 47- and 77-days post hatching. At day 56, the percentage of mature animals showed a two-phase response in both Cu form exposures. CuONM caused a concentration-dependent decrease in the percentage of adults up to 300 mg Cu/kg soil DW, while it increased at 400 mg Cu/kg soil DW. On the other hand, exposure to CuCl_2_ caused a U-shape response, with an initial decrease on time and a later increase at the higher concentrations (Fig. 1d). After 77 days of exposure, all the earthworms independently of the treatment had reached maturity.

The cocoon production, hatchability and juvenile production was affected concentration-dependently by both Cu forms. CuCl_2_ was more toxic to cocoon production than CuONM (EC10: CuONM = 92(7−177) mg/kg soil DW and CuCl_2_ = 47(11−83) mg/kg soil DW, Table 2). On the other hand, juvenile production was more affected by CuONM than by CuCl_2_ (EC10: CuONM = 62(21−103) mg/kg soil DW and CuCl_2_ = 47(12−83) mg/kg soil DW, Table 2). Regarding hatchability, CuONM exposure caused a decrease in hatching EC10 CuONM = 265(184−346) mg/kg soil DW, Table 2), while no effect of CuCl_2_ was detected. Juvenile production was the most sensitive reproductive endpoint with the lowest EC values (Table 2).

**Fig. 2:**
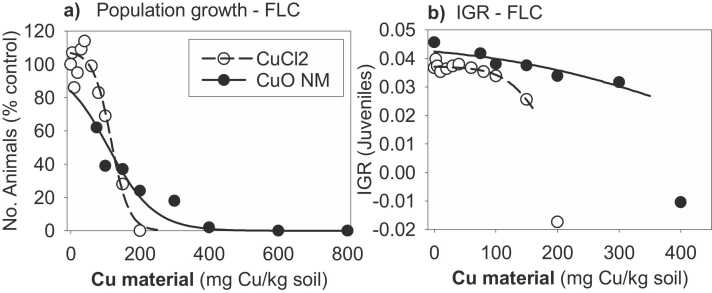
(a) Population growth (no. of organisms (% of control) at the end of the life cycle test) and (b) Instantaneous growth rate of the population based on juveniles after the exposure to CuONM (10 concentrations) and CuCl_2_ (12 concentrations). Concentrations are reported as mg Cu/kg soil DW. Symbols: closed/black circles refer to CuONM and open/white circles to CuCl_2_.

Population growth decreased concentration-dependently after exposure to CuONM (Fig. 2a). Regarding CuCl_2_, concentrations below 60 mg Cu/kg soil DW increased population growth, but higher concentrations had a negative effect on the number of produced organisms. Concerning the IGR of juveniles, it decreased linearly with increasing Cu concentration in soil, reaching negative values after exposure to 400 mg Cu/kg soil DW. CuCl_2_ exposure also caused a concentration-dependent decrease in IGR, but nearly all the exposures rendered positive values (Fig. 2b). The exceptions were the highest exposure concentration from each of the materials exposures.

### 3.3 OECD reproduction test

**Fig. 3:**
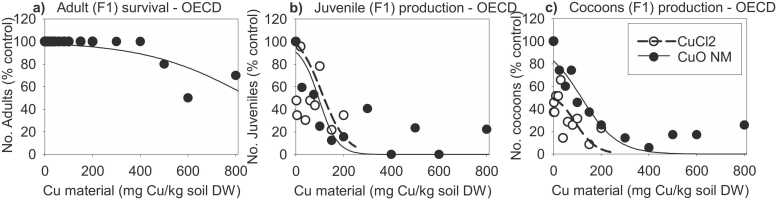
(a) Survival, (b) reproduction and (c) cocoon production of adult earthworms (% control) from the standard OECD reproduction test after exposure to CuONM (10 concentrations) and CuCl_2_ (12 concentrations), Soil concentrations are reported as mg Cu/kg dry soil. Survival symbols in CuCl_2_ are not visible in graph as they are at the same place as for the CuONM until 200 mg Cu.

During the standard OECD reproduction test, no mortality occurred in the exposure to CuCl_2_, while for CuONM survival decreased at concentrations higher than 500 mg Cu/kg soil DW (Fig. 3a). Juvenile and cocoon production also decreased in a concentration-response manner, with EC50 values of around 100 mg Cu/kg soil DW for both exposures (Fig. 3b & c, Table 2).

Bioaccumulation was measured in CuONM exposed organisms [the CuCl_2_ samples was lost so could not be measured] (Fig. S3). For both the FLC and the standard OECD test there was an increase in internal Cu concentration with in increasing external total Cu concentration. The FLC test organisms’ had a higher content at lower exposure concentration, especially at intermediate exposure concentrations (100–150 mg Cu/kg soil) there was a much higher internal concentration. No such pattern was observed in the organisms from the standard OECD test, where a monotonic dose-response occurred.

## 4 Discussion

The multi-endpoint FLC approach with *E. fetida* provided comprehensive information regarding CuONM and CuCl_2_ effects on juvenile survival, growth, and maturity, besides survival and reproduction. From these measured endpoints we derived population level effects for exposure to both Cu forms. Both Cu forms caused negative effects on the different stages, as in agreement with previous results (Heckmann et al., 2011, Velicogna et al., 2021). The results provide additional information to the standard OECD test, showing that Cu exerted its effect on juvenile mortality and cocoon production – which is not seen in the standard OECD test.

The active Cu in soil solution (i.e., Cu^2+^) constituted less than 0.007% in both exposures, but the total Cu in the soil-solution was 2 − 3 fold higher for CuCl_2_ than for CuONM spiked soils, i.e., when comparing exposure concentrations between 150 and 300 mg Cu/kg. This, to an extent, reflects the higher toxicity of the CuCl_2_, at comparable concentrations. We observed a gradual decrease over time for the soil solution Cu concentration for both CuCl_2_ and CuONM (Mendes et al., 2018), which agrees with a higher toxicity in the earlier phases of the exposure. The decrease in soil solution Cu over time is in line with observations by Joko et al., 2021, who also showed a gradual decrease up to 840 days. This decrease must be due to complexation with the soil components.

In fact, juveniles were especially susceptible during the first 28 days of post-hatching development, with no further mortality recorded after that period. The higher sensitivity during the juvenile stage as to survival could be due to a fast uptake via dermis in juveniles (i.e. larger surface area than when adults) (Bicho et al., 2017a, Spurgeon et al., 1994). The sensitivity of the juvenile phase is in agreement with previous studies with CuCl_2_ exposure, with other worm species like *Dendrobaena octaedra* (Bindesbol et al., 2007), *Lumbricus rubellus* (Spurgeon et al., 2004), and *Enchytraeus crypticus* (Bicho et al., 2017a).

On the other hand, the juvenile growth rate (i.e., of the surviving *E. fetida*) was not correlated with total soil concentrations of CuONM or CuCl_2_, which is unlike previous observations where a negative impact on growth was described at high concentrations (Bicho et al., 2015, Bindesbol et al., 2007, Spurgeon et al., 1994). The fact that we did not see a correlation could be because of a concentration-dependent mortality (in both CuONM and CuCl_2_ exposures) which resulted in a lower density and higher food availability for the surviving animals [despite that we assumed food to be ad libitum]. This would explain an enhanced growth (Domínguez and Edwards, 1997, Wright, 1972) and agrees with that the maximum weight of the earthworms was negatively correlated with the survival. Also related to juvenile growth is another important endpoint, i.e., “time to maturation” (Calow and Sibly, 1990, Vangestel et al., 1991), and here we also did not observe any monotonic relationship to the exposure concentration, instead there was a decrease at lower concentration and an increase at higher CuONM, possibly a hormesis effect. This is similar to Bicho et al., 2017a who observed few mature organisms at day 22–25 with increasing exposure concentrations, both for CuCl_2_ and for CuONM, for *Enchytraeus crypticus*.

Our results do not show whether the two Cu forms caused a difference in effect on hatchability in the FLC test. This no effect was observed for CuCl_2_ at the concentrations tested (maximum 200 mg Cu/kg soil DW) and for CuONM the effect started at exposure concentrations higher than 200 mg Cu/kg soil DW. Previously, we observed a stronger effect of CuONM compared to CuCl_2_ in *Enchytraeus albidus* (Amorim and Scott-Fordsmand, 2012), here juvenile toxicity of CuONM was stronger than the corresponding salt. Bicho et al., 2017a using *E. crypticus* did not observe any effect of CuONM on hatchability, but did so for CuCl_2_.

The overall population level parameters determined through the life-cycle test, i.e., the IGR and population growth clearly indicate the population decline produced by the exposure to CuONM and CuCl_2_. Moreover, as seen above, the negative IGR for both CuONM and CuCl_2_ reflected differences in sensitivities in cocoon production and juvenile survival. The population growth exhibited a clear concentration-dependent population decay caused by CuONM exposure, being completely inhibited at concentrations higher than 400 mg Cu/kg soil DW and 150 mg Cu/kg soil DW for CuONM and CuCl_2_ exposures respectively. These results are in agreement with the observation for *E. crypticus* (Bicho et al., 2017a).

Compared to the OECD standard test, the FLC provided a more complete evaluation of toxicity to *E. fetida* allowing for a better understanding of how toxicity acts on the population. This highlights the importance of addressing effects at different developmental stages and not only in adults. From a hazard assessment point of view (e.g. for use in Species Sensitivity Distributions (Scott-Fordsmand and Jensen, 2001; Scott-Fordsmand et al., 1996)) results from both tests can be used to calculate the Hazard Concentration. However, while both the life cycle test and the OECD produce ECx values for the reproductive output, these “the same” endpoints do not represent comparable population effects. The exposure history of the adults in the two tests was different. The soil from the FLC had earthworm activity and aging for 77 days prior to starting the equivalent OECD standard test, which means that there is also an aging effect compared to a traditional test (Diez-Ortiz et al., 2015, Josko et al., 2019, Liu et al., 2005, Scott-Fordsmand et al., 2004, Sizmur and Hodson, 2009, Waalewijn-Kool et al., 2013). Hence, care should be taken when pooling data in statistical extrapolation methods.

## 5 Conclusion

To conclude, the multi-endpoint approach of the FLC test used in the present study indicated that it is a suitable test method for assessing the toxicity that emerging pollutants like nanomaterials could cause to *E. fetida.* The information they provide is more complete to perform a correct and improved toxicity evaluation. The population growth was a reliable indicator of the impact of CuONM and CuCl_2_ on earthworms. The life-cycle test enabled the detection of the most vulnerable developmental stages and elucidate different life stage sensitivities between the two Cu exposures.

## Declaration of Competing Interest

The authors declare that they have no known competing financial interests or personal relationships that could have appeared to influence the work reported in this paper.
