# Supplementary material for: Full life cycle test with Eisenia fetida - copper oxide NM toxicity assessment
Source: Ecotoxicol Environ Saf. 2022 Aug;241:113720. doi: 10.1016/j.ecoenv.2022.113720 (PMC9289749; doi:10.1016/j.ecoenv.2022.113720)
Supplement: Supplementary file 1 — Supplementary material [file mmc1.docx]

**Full life cycle test with *Eisenia fetida* - Copper NM toxicity assessment**

J.J. Scott-Fordsmand^1^, A. Irizar^1^, M.J.B. Amorim^2^

^1^Department of Bioscience, Aarhus University, Vejlsøvej 25, DK-8600 Silkeborg, Denmark

^2^Department of Biology & CESAM, University of Aveiro, 3810-193 Aveiro, Portugal

*Corresponding author:

Janeck J. Scott-Fordsmand, Department of Bioscience, Aarhus University, Vejlsøvej 25, DK-8600 Silkeborg, Denmark. E-mail: [jsf@ecos.au.pt](mailto:jsf@ecos.au.pt);

**Figure S1.** Results from the Full Life Cycle test on *E. fetida* exposed to CuONM and CuCl_2_ (mg Cu/kg dry soil) for the various endpoints: (a) juvenile survival (day 77), (b) cocoon production, (c) hatchability, (d) adult survival (day 105), and (e) juvenile production (day 133).

**Growth curves**


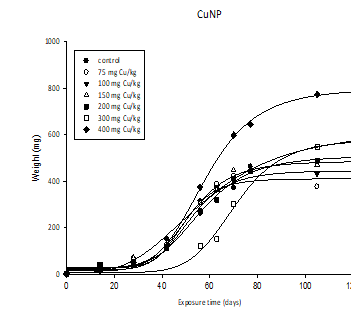

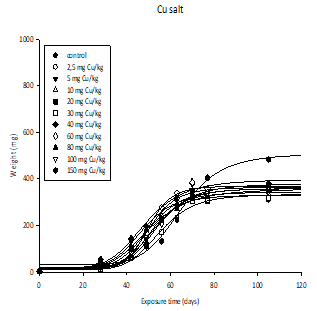


**Figure S2.** Growth curves of *E. fetida* exposed to different concentrations of CuONM (a) and CuCl_2_ (b) (mg Cu/kg dry soil) within time (>100 days).

Juvenile growth with the logistic model fit over the data. No apparent monotonic concentration-response trend was observed as to the growth rate and maximum weight, although it is clearly visible that individual growth curves and final weight differed.

**Bioaccumulation**

**Figure S3**. Bioaccumulation of CuONM (mg Cu/kg soil DW) in *E. fetida* adults after exposure via a Full Life Cycle test (105 Days) and the standard OECD test (28 days).
